# Recent advances of metal-based nanoparticles in nucleic acid delivery for therapeutic applications

**DOI:** 10.1186/s12951-022-01650-z

**Published:** 2022-11-24

**Authors:** Ashish Ranjan Sharma, Yeon-Hee Lee, Altanzul Bat-Ulzii, Manojit Bhattacharya, Chiranjib Chakraborty, Sang-Soo Lee

**Affiliations:** 1 Institute for Skeletal Aging and Orthopedic Surgery, Hallym University-Chuncheon Sacred Heart Hospital, Chuncheon-si, 24252 Gangwon-do Republic of Korea; 2 Department of Zoology, Fakir Mohan University, Vyasa Vihar, Balasore, Odisha 756020 India; 3 Department of Biotechnology, School of Life Science and Biotechnology, Adamas University, Ba-rasat-Barrackpore Rd, Kolkata, West Bengal 700126 India

**Keywords:** Nanomaterials, Nucleic acid, Drug delivery, Metal nanoparticles

## Abstract

Recent efforts in designing nanomaterials to deliver potential therapeutics to the targeted site are overwhelming and palpable. Engineering nanomaterials to deliver biological molecules to exert desirable physiological changes, with minimized side effects and optimal dose, has revolutionized the next-generation therapy for several diseases. The rapid progress of nucleic acids as biopharmaceutics is going to alter the traditional pharmaceutics practices in modern medicine. However, enzymatic instability, large size, dense negative charge (hydrophilic for cell uptake), and unintentional adverse biological responses—such as prolongation of the blood coagulation and immune system activation—hamper the potential use of nucleic acids for therapeutic purposes. Moreover, the safe delivery of nucleic acids into the clinical setting is an uphill task, and several efforts are being put forward to deliver them to targeted cells. Advances in Metal-based NanoParticles (MNPs) are drawing attention due to the unique properties offered by them for drug delivery, such as large surface-area-to-volume ratio for surface modification, increased therapeutic index of drugs through site-specific delivery, increased stability, enhanced half-life of the drug in circulation, and efficient biodistribution to the desired targeted site. Here, the potential of nanoparticles delivery systems for the delivery of nucleic acids, specially MNPs, and their ability and advantages over other nano delivery systems are reviewed.

**Graphical Abstract:**

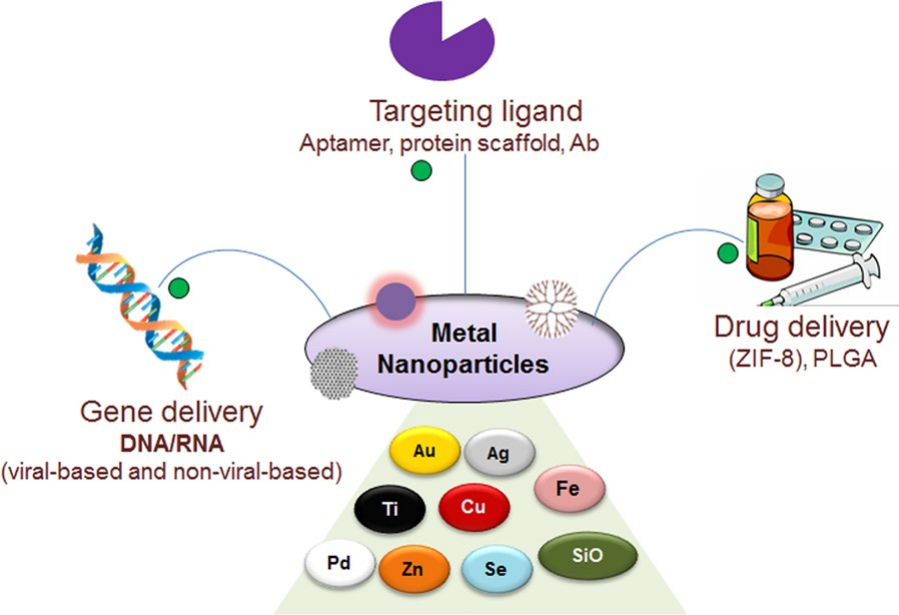

## Introduction

Nanotechnology enables technology to deal with nanometer-sized objects and to apply them in an array of applications. The concept of nanotechnology was first introduced in 1959 by Richard P. Feynman [1]. Nowadays, nanotechnology is becoming more advanced and refined in scientific terms, and is being applied to various biological applications. For example, nanotechnology is contributing to multiple aspects of diagnosis and therapies for several diseases [2].

**Fig. 1:**
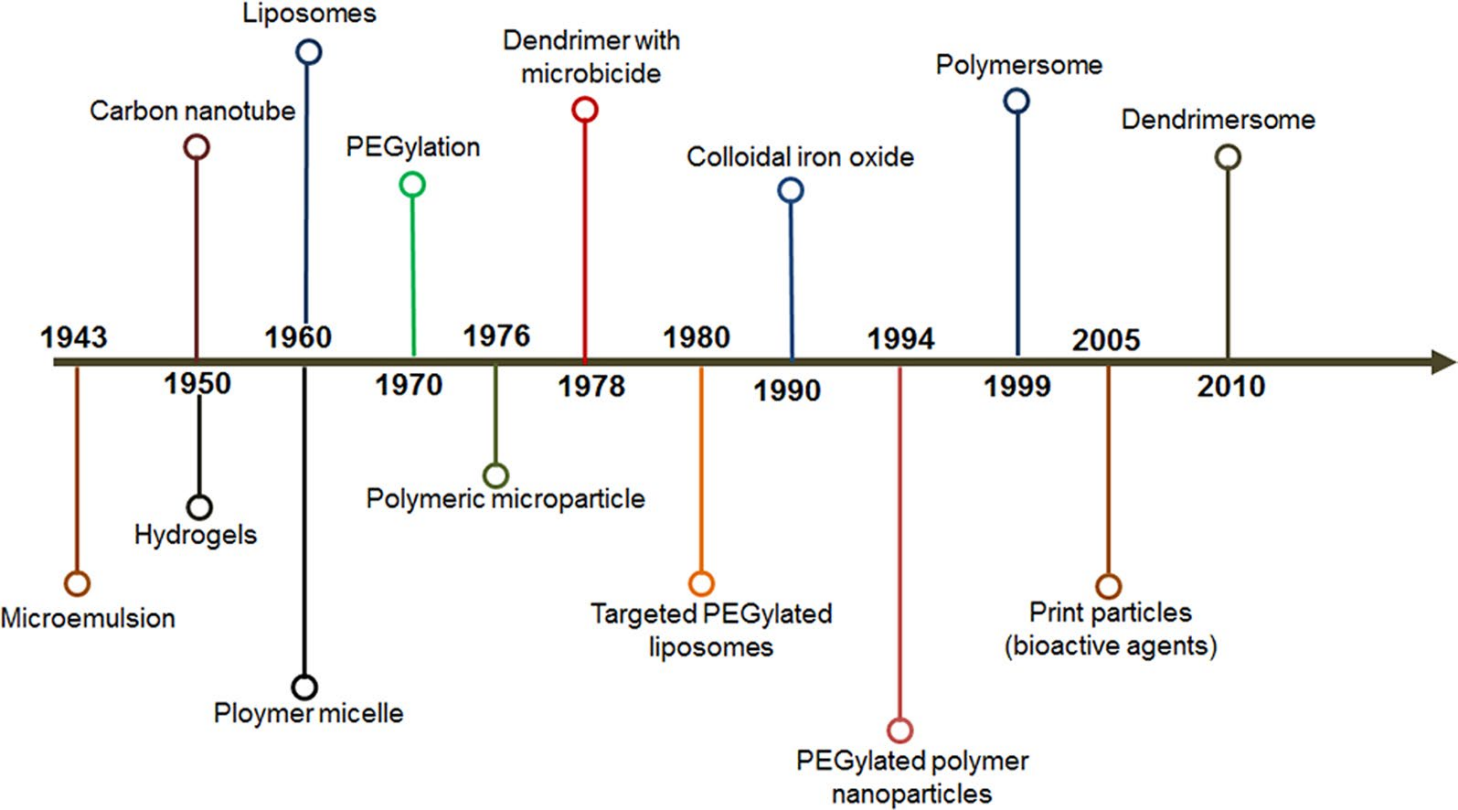
The timeline shows the development of several NPs

Nanoparticles (NPs) are the fundamental constituents responsible for nanotechnology’s applicative properties. NPs have been studied for decades due to their unique chemical and physical properties such as: (a) small size, less than 100 nm at least; (b) size-dependent immense surface area per unit volume; (c) high proportion of atoms in the near-surface layers, and (d) an ability to exhibit quantum effects [2, 3]. Their overall shape can also exhibit the morphological diversity of 0D, 1D, 2D, or 3D, which means disks, platelets, spheres, and tubes [4]. NPs have a tremendous chemical variety that can be present in several forms: organics or biologicals, carbon materials, polymers, semiconductors, and inorganics, including metals and metal oxides [5]. A timeline representing the development of various types of the NPs is shown in Fig. 1. NPs are synthesized through several means based either on the gas, liquid, or solid-phase approaches and can be surface functionalized to activate and stabilize according to the needs of specific applications [6]. Some of the synthesized NPs are environmentally friendly, producing more homogeneously distributed NPs, and readily biodegradable [7]. NPs have been researched for various biomedical applications like delivery systems, bio-sensing, imaging, and antimicrobial applications [8].

Metal-based NPs (MNPs) have been applied in different fields due to their unique properties. They have many advantages due to their unique characteristics, but it is also a fact that they have disadvantages due to shape, size, composition, surface area, and charge. In particular, they are widely used as carriers of drugs, biomolecules, and genes in medical therapy areas. MNPs have been used for imaging and active or passive tumor cell targeting as carrier and contrast agents [9]. The development of these MNPs opens the way for novel drug delivery platforms, site-by-site targeting, and gene delivery [10]. For example, gold NPs (AuNPs) are known gene carriers for the treatment of cancer because of their excellent biocompatibility with biomolecules like nucleic acid [11]. Silver NPs (AgNPs) with an enhanced gene-binding affinity as efficient therapeutic gene carriers offer the potential gene delivery system to treat various diseases such as cancer [12, 13]. Iron oxide NPs (IONs) have been suggested as a therapeutic agent and gene carrier for breast cancer treatment by utilizing their superparamagnetic, highly biocompatible, and biodegradable properties [14]. The practical potency of MNPs also highlights their potential as new, improved modalities for future therapeutic agents [15]. Considering the emerging role of MNPs in the delivery of nucleic acids, we have tried to recapitulate the potential and future of MNPs as therapeutics for human diseases.

## NPs for nucleic acid delivery

The biomaterial transfection agent must have adequate ability to bind and encapsulate the nucleic acid. Nucleic acids are negatively charged, thus biomaterials with a positive charge are often preferred. For profitable intracellular delivery of nucleic acids, the NPs must be in a position to enter the cytoplasm safely and efficiently [16]. Whilst siRNA should be launched in the cytoplasm, DNA should be trafficked to the nucleus, the main web page of function [17]. In 2011, Zheng et al. described that the best siRNA transport gadget should be “biodegradable and biocompatible” [18, 19]. Meanwhile, a multitude of new formulations that condense siRNA into nano-sized particles appropriate for intracellular transport with the aid of endocytosis has been examined in vitro, and a smaller quantity of nano-formulations have been assessed for in vivo transport of siRNA [19]. Delivery processes are commonly divided in viral and non-viral methods. Viral transport methods, such as adenoviruses, retroviruses, and adeno-associated viruses, are advantageous in delivery effectivity; however, frequent use may result in safety issues due to immunogenicity and resistance to repeated resistance infection. Therefore, emerging non-viral delivery techniques for siRNAs has been and will proceed to be the center of attention of significant research efforts [20–24].

**Fig. 2:**
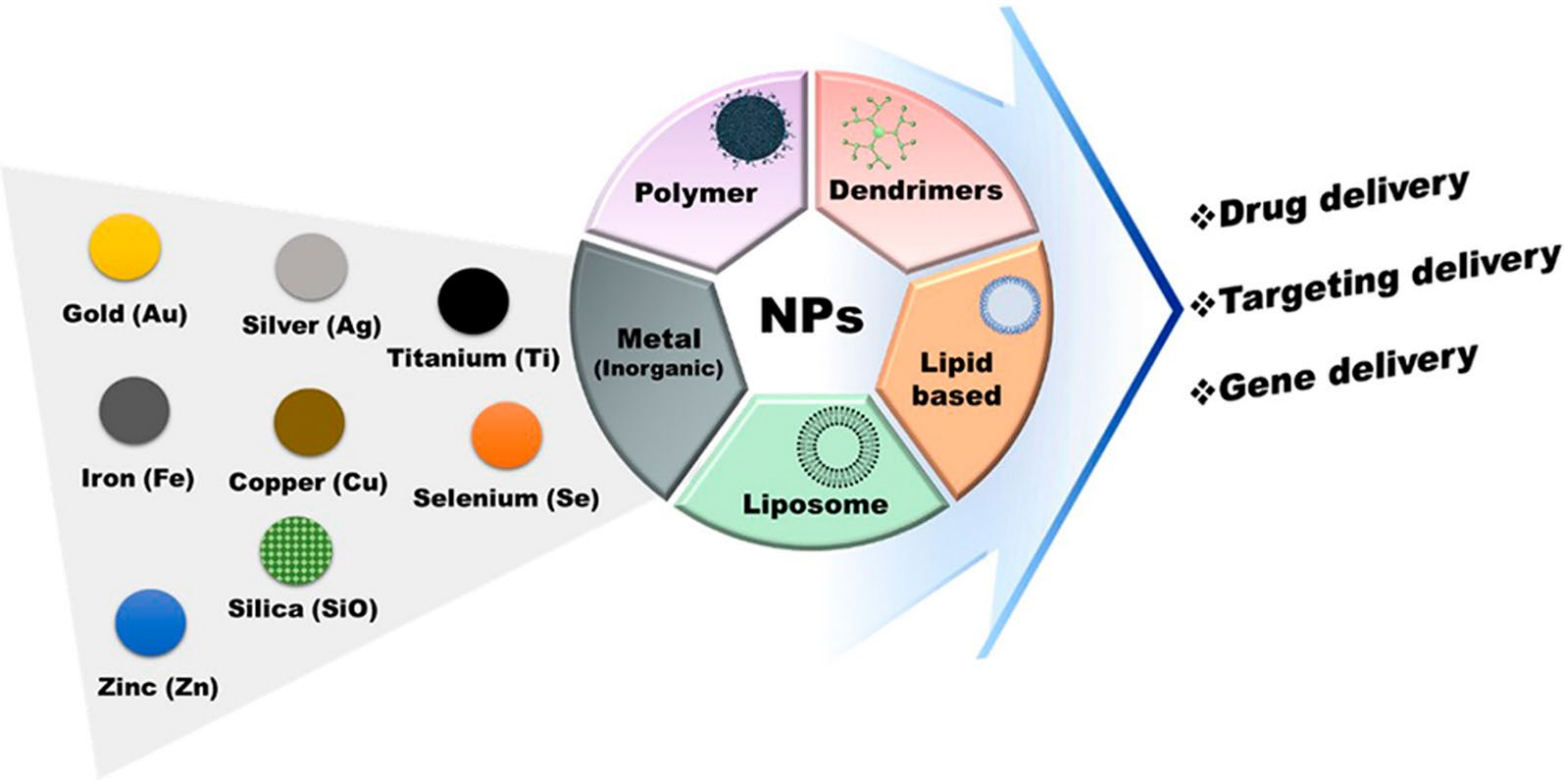
Type of NPs for the range of delivery system

NPs are composed of different types of material, including polymers, metals, proteins, lipids, and semiconductors. With the use of breakdown or buildup of systematizing techniques, NPs are synthesized in different forms and characterized regarding their properties [25, 26]. Targeting tumors nanocarrier structures can be divided into 3 categories: natural vectors (e.g., lipid-based NPs and polymer-based NPs), inorganic vectors (e.g., magnetic NPs, AuNPs, and quantum dots), and hybrid vectors (e.g., theranostic NPs that incorporate each natural and inorganic supplies) [27]. Figure 2 represents different types of NPs as carriers and their applications. The advantages and disadvantages of NPs used as gene carriers are summarized in Table 1.

**Table 1:** Advantages and disadvantages of several NPs for nucleic acid delivery

Types	Advantages	Disadvantages	References
Liposome	▪ Self-assemble as multilamellar cationic liposome-nucleic acid (lipoplex) ▪ Efficient and functional delivery of nucleic acids	▪ Inconsistent storage characteristics ▪ Colloidal instability ▪ Instability in organic fluids ▪ Unpredictable efficacy ▪ Cytotoxicities	[29–32]
Lipid	▪ Common nanocarriers ▪ Tolerable toxicity and low immunogenicity ▪ Safety ▪ Biodegradability and biocompatibility	▪ Difficulty of topical administration	[31–41]
Peptide	▪ Biodegradability ▪ Biocompatibility	▪ Not able to cross the cell membranes ▪ Effortlessly degraded with the enzymes	[42–45]
Polymer	▪ Biodegradable and appearance modifiable ▪ Extension of the effectiveness ▪ Non-carcinogenic ▪ Non-mutagenic and non-immunogenic	▪ Non-safety ▪ Toxicity	[46–50]
Dendrimers	▪ Assembles a number of proteins ▪ Attaining a range of functions ▪ Low toxicity ▪ Intracellular release and bioactivity of the delivered proteins	▪ Instability ▪ Weak interactions between gene-dendrimer ▪ Toxicity	[51–60]
Micelle	▪ Self-assembly ▪ Condensation and safety of nucleic acids ▪ Solubilization of hydrophobic and hydrophilic drugs or genes ▪ Safer toxicity	▪ Disassociate below the critical micelle forming concentration (CMC) ▪ Low stability as gene delivery	[61, 65]
Inorganic (metallic)	▪ Low cytotoxicity ▪ Diagnostic abilities via imaging ▪ Outstanding pharmacokinetic ▪ Crossing biological barriers ▪ Adjustable size ▪ Storage stability	▪ Difficulty in synthesis ▪ Biologically harmful	[43, 66–68]

### Liposome

Simple cationic liposomes that engage with nucleic acid (lipoplex) can surprisingly act as effective functional delivery for nucleic acids to the lungs [28, 29]. However, once simple cationic liposome structures are formulated in aggregation with nucleic acids, they undergo extensive forms and demonstrate colloidal instability, persistent instability in organic fluids, inconsistent storage characteristics, and inconsistent efficacy, leading to undesirable cytotoxic results [30]. Wang et al. synthesized Poly(lactide-co-glycolide)/folate coated PEGylated polymeric liposome core–shell NPs (PLGA/FPL NPs). This delivery system consisted of a hydrophobic PLGA core and a hydrophilic lipid shell for the drug and gene co-delivery system. The complex was successfully delivered to MDA-MB-231 breast cancer cells. They concluded that core–shell NPs with a concentration under 10 mg/ml caused no significant cytotoxicity against this cell line [31].

### Lipid-based NPs

Lipid-based NPs (LNPs) are the most widely used and most studied nanocarriers for cancer therapy [32]. RNAi-lipid-based nanocarriers are able to provide security against serum nucleases enabling prolonged circulation, leading to a greater uptake by the target tissue [33]. Lipid nanocarrier’s enormous advantages are their excellent biodegradability and biocompatibility. Likewise, most LNPs exhibit bearable toxicity and low immunogenicity [34]. In many previous anticancer immunotherapeutic methods, lipid-based nanovectors have empowered the integration of mRNA-based science, such as monoclonal antibodies, Chimeric Antigen Receptor (CAR) cell therapy, and therapeutic vaccines [35]. Developing the LNPs delivery method has enabled the scientific translation and approval of the first siRNA drug to inhibit pathogenic protein manufacturing in hepatocytes [36]. Some LNPs and polymer NPs are potentially toxic at high doses, and usually have low delivery effectiveness than other systems with higher stability and appreciable surface area [37, 38]. Kaczmarek et al. developed hybrid polymer-lipid nanoformulations to act as a nanocarrier for functional delivery of the mRNA in HeLa cells (cervical cancer cells), as well as to the primary mouse lung cells. Importantly, these data demonstrated an association between serum stability and in vivo efficacy [39]. Sun et al. reported a safe and efficient delivery system for genetic eye disease therapy. They synthesized NPs including multifunctional lipid ECO (1-aminoethyl)iminobis[N-(oleicylcysteinyl-1-amino-ethyl)propionamide], a hybrid fourth generation nano-globule and plasmid DNA (pDNA) with core shell dendrimers, for delivering gene into (Retinal Pigment Epithelia (RPE)) cells in vivo. They also used this delivery system in ARPE-19 cells (human retinal pigment epithelium cells) in vitro. They mentioned that low N/P ratio is suitable for efficient and safe gene delivery and low N/P ratio G4/ECO/pDNANPs showed low cytotoxicity and high stability. Under both conditions (in vitro and in vivo) NPs mediated gene transfection was efficient [40].

### Peptide

Peptides have great biodegradability and biocompatibility, therefore they have been a very optimal organic material of choice [41]. However, peptides do not have the ability to cross cell membranes and are effortlessly degraded by enzymes. These short-comings restrict peptides from translation from experimental studies to medical applications [42]. Veiman et al. developed negatively charged bioactive NPs using the new modified cell-penetrating peptide, PepFecs (PF14), they designed along with pDNA. Their research work concluded that negatively charged particles stay longer in the systemic circulation than positive surface charges. PF14/pDNA complex transfection efficiency was also high in adherent cell lines such as Chinese Hamster Ovarian (CHO) cells, U2OS (human bone cells), U87 (human astrocytes), and HEK293 (human embryoinic kidney) cells [43]. Zarei et al. designed a gene delivery system based upon Low Molecular Weight Protamine (LMWP). They prepared Peptide-pDNA (PD) and LiposomePD (LPD) nanocarriers from VV45 and VV32 peptides, and pDNA was encoding the Green Fluorescent Protein (GFP). In the A549 cell line (lung cancer), the transfection effect of LPD was higher than PD. The cytotoxicity depended on the C/P ratio, and the C/P ratio significantly increased cell viability. At C/P ratio 1, there was no cytotoxicity [44].

### Polymer NPs

Polymer NPs are biodegradable and appear modifiable. These biodegradable polymer NPs shield and supply the drugs and RNAs, with subsequent degradation of the polymer matrix over a length of time, and have the functionality to supply more than one drug in one carrier. Polymer NPs can reach passively and actively the tumor cells. Drugs and RNAs delivered along polymer NPs can extend the drug’s effectiveness in contrast to direct delivery [45]. Engineered polymer NPs are regarded to be safe, non-toxic, non-carcinogenic, non-mutagenic, non-immunogenic, relatively simpler to synthesize, and appear modifiable. However, the safety and toxic consequences of these polymer NPs have not yet been solved to date, raising some concerns. Nanotoxicology is gaining great interest in learning about the security assessment of nanomaterials that are deliberate for use in more than a few biomedical applications [46, 47]. Li et al. revealed that cationic polymers containing disulfide also had very low cytotoxicity at a high weight ratio of polymer/DNA and were efficient for gene delivery in U2OS cells [48]. Witzigmann et al. developed a polymer-peptide hybrid-based DNA system for gene delivery. They used the biocompatible synthetic polymer poly(2-methyl-2-oxazoline) (PMOXA) and the peptide block poly(aspartic acid) (PASP), modified with diethylene-triamine (DET). The PMOXA-b-PASP-DET system was complexed with pDNA and delivered genes efficiently to HEK293 and HeLa cells without significant cytotoxicity. They concluded that the Polymer–Peptide Hybrid (PPH) system has many chemical modification options and is beneficial for in vivo applications [49].

### Dendrimers

Poly(amidoamine) (PAMAM) dendrimers are the earliest and most extensively used polymers. They have layered 3-dimensional constructions and are substantially used for scientific applications. To attain a range of functions such as enhancing concentrated ability, regulating solution behavior, and lowering toxicity, the adaptable surface conjugation of PAMAM enable bind to or soak up unique sorts of reagents [50, 51]. The dendrimer successfully assembles a number of proteins into NPs, and the delivered proteins may become bioactive after intracellular release [52]. PAMAM dendrimers possess surprisingly hydrophobic indoors enabling encapsulation of hydrophobic chemotherapeutic agents and enhancing both their water solubility and bioavailability. Moreover, they provide a hydrophilic floor with free amine moieties that let in each siRNA grafting onto the surface and enable endosomal escape through the proton sponge effect. Thus, each siRNA and chemotherapy drug can be delivered into the cytoplasm where they are needed. Ghaffari et al. reported a combined curcumin (Cur) delivery system and Bcl-2 siRNA effect on cancer cells: a PAMAM dendrimer nanocarrier that was used to maximize the increase of apoptosis. The PAMAM-Cur/siRNA complex’s anti-proliferative activity was higher than the one of the PAMAM-Cur complex. They concluded that PAMAM-Cur with Bcl-2 siRNA complex produced a more effective anticancer agent for the HeLa cell line and cervical cancer [53]. All the other properties of a compound and its toxicity are directly connected to its structure. Some elements that compose a dendrimer, such as core, branch, and surface groups, regulate toxicity levels.

Dendrimers react with biological membranes, leading to a particular disruption and cell death [54]. Nam et al. reported a new synthesized PAM-ABP system. Arginine grafted bio-reducible polymer (ABP) has high transfection efficiency but low molecular weight. Hence they synthesized ABP with PAMAM dendrimer (PAM-ABP) to overcome the low molecular weight. In the MCF-7 breast cancer cell line and A549 lung adenocarcinoma epithelial cell line, PAM-ABP delivered pDNA efficiently. They concluded that PAM-ABP had superior efficacy and maybe the efficacy compared to ABP is a favorable delivery system for gene therapy [55]. In another study, Kim et al. also reported that PAM-ABP delivered genes in human mesenchymal stem cell (hMSC) and showed low toxicity [56]. Yu et al. used PAMAM dendrimer for delivering siRNA to silence Hsp27 in human prostate cancer cells. In their experiment, cytotoxicity was not observed. Both in vivo and in vitro gene silencing were significantly achieved [57]. Bae et al. developed an apoptin gene delivery system using ornithine modified PAMAM (PAMAM-O) dendrimer. They tried it in HepG2 cells and dermal fibroblasts. In both cell types, cytotoxicity was low, but the transfection effect of PAMAM-O was significantly higher in HepG2 cells. PAMAM-O/Flag-apoptin complex showed cell apoptosis in HepG62 cells but not in dermal fibroblasts. Due to low cytotoxicity and high transfection effect, the PAMAM-O/Flag-apoptin complex could be a promising candidate for gene delivery in HepG2 cells [58].

### Micelles

The borderline between micelles and polyplex NPs, called “micelle-like NP (MNP)” provides wonderful facets, including self-assembly, encapsulation, security of nucleic acids, extended mobile affiliation, gene transfection solubilization of hydrophobic and hydrophilic drugs, and safer toxicity profile. The micelles' steadiness is commonly given as its Critical Micelle Concentration (CMC), described as the attention of a monomeric amphiphile [59]. Despite the advantages, their low stability, while in conflict with environmental changes, is a basal limitation as drug or gene delivery carriers. If the concentration is under the CMC, micelles can separate, which is characteristically observed when micelle formulations are administered into the blood [60]. Polyplex Micelles (PM) have a high surface charge and gene-bonding affinity. PM safely carries the mRNA to cells and mouse lungs [61]. Polyplex Nanomicelles (PNM) efficiently deliver Runx1 mRNA to primary BMSCs and are shown to play a role in alleviating the progress of vertebral disc degenerative disease after injection of PNM-Runx1 to rat disc degeneration model [62]. Dbait is a small double-stranded DNA molecule that is utilized as a radiosensitizer. Developed microenvironment-responsive micelle (ch-Kn(s–s)R8-Angiopep-2/Dbait) significantly inhibited the growth of U251 cells in vitro and enhanced the efficacy of radiotherapy [63]. Cis-aconitate-modified chitosan-g-stearic acid (CA-CSO-SA) micelles were successfully uptaken into the cells by clathrin-mediated endocytosis, caveolae-mediated endocytosis, and macropinocytosis, and escaped from the endosome. Also, CA-CSO-SA/pDNA complex has higher efficiency of pDNA transfection than the CSO-SA/pDNA complex in HEK-293 cells [64].

### Inorganic NPs

As a workable miRNA and siRNA delivery mechanisms, non-viral inorganic NPs are improving since they offer extra advantages in contrast to typical viral vectors, such as adjustable size, storage stability, focus on targeting directly towards the favored site of function, enabling cross biological barriers, outstanding pharmacokinetic and toxicity profiles, and granting concurrent diagnostic availabilities via imaging [53]. In the last decades, inorganic NPs such as gold, iron oxide, silica, and quantum dots have emerged as alluring non-viral gene vectors [65]. They are favorable in many applications since they are more consistent with less cytotoxicity against other MNPs, and, consequently, have started to play a role in areas such as cancer diagnosis and drug or gene delivery [66]. MNPs have a few disadvantages: particles’ instability, biologically harmful, impurity, difficulty in synthesis, and explosion (some MNPs are thermodynamically unstable). These may lead to poor corrosion resistance, and maintaining the structure will be difficult. There can be high chances of impurity since MNPs are highly reactive. While synthesizing MNPs, nitrides, oxides, etc., can be aggravated by the unclean environment. Some researchers reported that nanomaterials are carcinogenic, toxic, and also, when translucent to the dermis, irritation can occur [67]. We have tried to recapitulate the potential of various types of MNPs for the delivery of nucleic acids and their ability to project them as therapeutic in the near future.

## Synthesis and characterization of MNPs

NPs open the prospective to develop a series of medical and biotechnology tools and techniques that may be used as safer, portable, cheaper, and easier to handle techniques for up to the sub-cellular levels. These are also used for a number of other purposes, from medical treatments to numerous branches of industrial production and materials of everyday use, as well as biological labeling and cure of human diseases [68, 69]. Presently, NPs have brought substantial attention to their therapeutic application and activities [70]. NPs can be synthesized by several methods, including chemical, physical and biological methods. Purposefully, several adverse effects have been linked with chemical synthesis methods due to toxic chemicals absorbed on the surfaces. Eco-friendly alternatives to physical and chemical methods are the biological ways of NPs synthesis through enzymes, microorganisms, fungus, plants, or plant extracts [48]. The development of different biological methods for NPs synthesis is rapidly improving into a significant branch of nanotechnology, especially MNPs, which have enormous applications in therapeutic approaches [71].

**Fig. 3:**
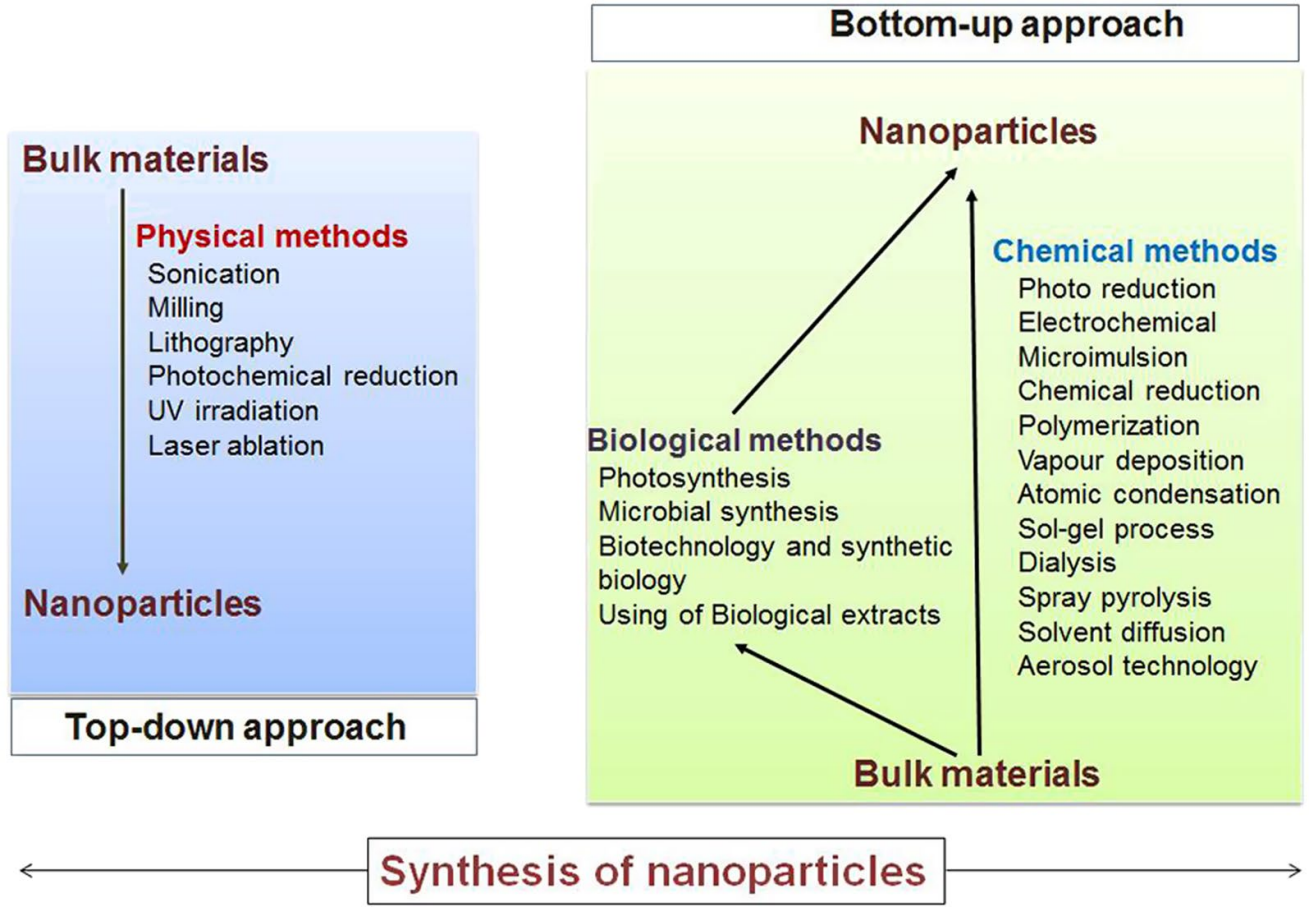
A brief outline of MNPs synthesis procedure

MNPs of variable sizes can be prepared by chemical as well as by physical methods. They show many interesting properties, the size-dependent metal to non-metal transition being the vital one [72]. MNPs coated with thiols can be organized into ordered 1, 2, and 3-dimensional structures, and these structures have potential applications as nanodevice [73]. In this context, the organization of arrays of MNPs with a fixed number of atoms should be significant [74]. Numerous methods are employed to prepare MNPs, categorized basically into two types: top-down methods and bottom-up methods, subject to the starting material of NPs preparation (Fig. 3) [75]. The synthesis of MNPs is followed by the characterization of NPs for specific attributes. TEM (transmission electron microscopy) evaluation is frequently used to analyze the occurrence of sphere-shaped MNPs (size range 3–45 nm). To analyze the composition, arrangements in crystal phase and range, various spectrometric techniques like UV–vis (ultra violet-visible light spectroscopy), FT-IR (Fourier-transform infrared spectroscopy), XRD (X-ray diffraction analysis), EDS (energy-dispersive X-ray spectroscopy), DLS (dynamic light scattering), and Raman are being performed [76]. Subsequently, the coherent characterization of NPs for nanomedicines contains its physicochemical depiction, assessment of pyrogenicity, biodistribution, and toxicity, which also comprises in vitro within the animal model and in vivo analysis [77]. Therefore, the unique chemical and physical properties of NPs at the molecular and atomic levels often interfere with the regular standard methods and compromise the reproducibility of their functions [78]. Thus, a selection of the synthesis methodology followed by the characterization of MNPs would play a crucial role in its application in nanomedicine.

## Application of MNPs for therapeutic delivery

Recently, notable progress has been made in NPs based delivery systems to living cells or animals. NPs mediated delivery of biomolecules or drugs has been extensively analyzed in vitro and in vivo studies. Owing to several advantages of MNPs, research has been favoring their functional ability to deliver drugs or nucleic acid molecules to targeted sites. This has generated enormous opportunities for NPs in biomolecule delivery to disease states [10]. Therefore, significant progress has been established in drug delivery, targeted delivery, and gene delivery for disease treatment [79].

### Drug delivery

With the advancement of drug delivery systems, numerous advantages are offered by NPs as therapeutic and diagnostic agents. One of them is that traditional drugs are not always manufactured as the optimal formulations, now available for oral or injectable administration [9, 80]. To protect from unwanted degradation and enhance efficacy, products comprising proteins or nucleic acids need a more advanced form of the carrier system. It is known that particle size (not including intravenous and solution) is directly related to the efficacy of most drug delivery systems [80]. Pertaining to their small size and increased surface area, therapeutic NPs demonstrate increased solubility, hence promoted bioavailability, additional capability to cross the blood–brain barrier (BBB), entry into the pulmonary system, and ability to cross the tight junctions of endothelial cells of the skin [81]. Particularly, NPs made from synthetic and natural polymers (biodegradable and non-biodegradable) are of particular concern as they can be easily modified for the drug targeted delivery. In addition to providing a controlled release of medication and improving bioavailability, these systems prevent the degradation of drugs from the endogenous enzymes through adaptation [82]. The development of new drug delivery systems might provide another advantage for pharmaceutical revenue for the companies. Due to innovative drug delivery systems, pharmaceutical companies try to develop new formulations for existing drugs [83]. Approaches like this will generate a great market force, pushing the advancement of more effective delivery methods for these novel formulations, making them favorable to the patients [84, 85].

MNPs for drug delivery are already being studied due to unique characteristic properties such as easy surface modification, high biocompatibility, and stability [86]. For instance, AuNPs can be used to deliver drugs to cancer cells by interacting with the folic acid Polyethylene Glycol (PEG)-amine on the surface of cancer cells [87]. Another example of MNPs is AuNPs-doxorubicin (DOX)-PEG NPs, which demonstrate good drug solubility and drug release, resulting in anticancer activity in a murine liver cancer model [88].

### Targeted delivery

The approach to deliver proper amounts of therapeutic agents for an extended period to the damaged area within the body is termed targeted therapy [85]. The development of therapeutic NPs having safe and enhanced compelling effects is therapy’s utmost goal. NPs are prone to aggregation and protein opsonization (protein binding to NPs surface as a tag for immune system recognition) as soon as NPs enter the bloodstream [89]. By phagocytosis or filtration in the liver, spleen, and kidney, the opsonized NPs could be taken away from the bloodstream. Due to the immune system’s rapid and non-specific clearance, the retention time is reduced, resulting in decreased bioavailability [90]. The modification of the surface can also change recognition abilities for targeted delivery. Furthermore, NPs with appropriate sizes can reach the targeted area comparatively easily, and gather for a longer period of time [91, 92]. Their dominant accumulation within a targeted area for action and targeted delivery refers to the effective direction of the therapeutic agents [92].

The agent-loaded system should be able to survive in the physiological system for a suitable time, evade the immune system, target only certain cells or tissues, and should release the loaded therapeutic agents achieving an effective targeted delivery [92, 93]. Presently, the NPs targeted delivery is widely considered in cancer management. Over 20% of the therapeutic NPs developed for anticancer applications are either already in clinics or under clinical evaluation [93]. Moreover, NPs-mediated therapy is being explored in some other fields such as infectious, neurodegenerative, autoimmune diseases, etc. [94]. By the near-infrared and Magnetic Resonance Imaging (MRI) mechanisms, Lee et al. [95] encapsulated oleic acid-coated FeO NPs in oleic acid-conjugated chitosan (oleyl-chitosan) to inspect the accretion of these MNPs in tumor cells. The purpose was to study the penetrability and holding consequences of the MNPs under in vivo conditions for analytical uses.

Target-based delivery approaches of MNPs for therapeutic purposes have significantly impacted science over the past few decades. In general, there are two types of targeted drug delivery: active targeted drug delivery (smart drug delivery)—based on ligands affiliation to receptors, and passive targeted drug delivery—based on the better permeability and retention effect within the cells or tissues [96]. A few passive targeted nanocarriers have been approved for clinical use, but none belongs to the actively targeted MNPs category [97]. The active targeting depends on ligand-receptor binding, which might to develop selective accumulation in the targeted sites, hence it can discriminate between healthy and disease affected tissues. Targeting could be achieved by aiming at the tumor cells, endothelial cells, the acidic environment of cancers, and the nucleus of the cells. Several studies suggest that the formulated NPs have to overcome numerous biological and physiological barriers. At the same time, their use as accurate delivering systems needs specific requirements like the extent of biocompatibility and topological chemistry, to prevent general interactions and achieve the specific binding to their targets [98, 99]. The selection between active or passive tumor targeting of MNPs shall largely depend on the features of the tumor cells and the chemical nature of the used drug. The drugs that do not have key issues regarding cellular penetration, and just need a simple encapsulation in a ‘stealth’ nanosystem (reaching the target passively) are adequate [100]. Lin et al. explained the active targeted strategy to kill or inhibit microbes by using active targeted drug delivery of NPs [101]. Conversely, Kunjachan et al. reported that the active targeted strategy should not be overestimated over passive targeting, as the retention time of drugs for the active targeted approach would be reduced easily in the studied animal model [102]. On the other hand, colloidal MNPs-based therapeutics are also an alternative and promising technique as organic therapies considering clinical treatment [103]. The MNPs can be used as a novel tool for theranostic applications such as molecular imaging, diagnosis, and therapeutic delivery of active agents to cancer-specific cells [104]. In an in vivo model, Gurunathan et al. reported the anti-angiogenic properties of silver NPs, validating and proving that these components could prevent VEGF-induced cell proliferation, migration, and formation of new blood microvessels [105].

### Gene delivery

Gene delivery is an essential part of gene therapy. Gene therapy is a technique that treats or prevents disease by inserting and delivering a gene such as exogenous deoxyribonucleic acids (DNA) or ribonucleic acid (RNA) into live cells instead of using drugs or surgery [106]. Therefore, gene therapy has great potential in modern medicine by developing treatments for underlying factors rather than the symptoms of the disease. However, delivery of a therapeutic gene to the targeted cells by crossing the plasma membrane still remains a limitation of gene delivery. For the highly efficient and safe delivery of nucleic acid into the targeted site, the study of the vector/carrier that is essential to carry the nucleic acid across the hydrophobic and negatively charged cell membrane is a prerequisite [107].

A successfully optimized vector/carrier that effectively compresses and offers stability until the nucleic acid moves to the targeted site in the cells, and safely transfers the nucleic acid into the nucleus by traversing the cell membrane, has to consider intracellular barriers (such as intracellular and nuclear uptake, endosomal escape, DNA release) and extra-cellular barriers (such as DNA degradation mechanisms for particle clearance) present in the cell system [106, 108].

There are two types of gene delivery: viral-based and non-viral-based delivery systems. Virus-based gene systems mainly use adenoviruses, retroviruses, and lentiviruses, which are unable to replicate (modify to replication-deficient), and are only capable of nucleic acid delivery and expression. The advantage is the constant expression of therapeutic genes but has limitations such as immunogenicity, toxicity, and lack of optimization [109]. Non-viral-based gene delivery systems are categorized as physical methods (such as microinjection, ultrasound, and hydrodynamic applications) and chemical methods (utilize natural or synthetic carriers) [110]. Liposomes, polymers, dendrimers, and inorganic materials are used for non-viral gene delivery [111]. This system has advantages such as low immune response, easy modification, and cell/tissue targeting. However, a major challenge is increasing the transfection efficiency of genes into the cells [111, 112].

Especially, MNPs can be modified with a variety of physical and chemical methods and can be conjugated with biological molecules. Thus, research on MNPs as carriers has growing interest and research is increasing in the chemical, biological and medical fields. For example, AuNPs can be modified according to the property of different biomolecules/ligands due to their soft surface. In addition, the stability of nucleic acid could be maintained over the long term compared to polymer NPs [113]. Methods like the glutathione (GSH)-mediated approach are being studied for nucleic acids delivery to cells and release from AuNPs [114].

## Application of various MNPs for nucleic acid delivery

Table 2 briefly shows several advantages and limitations of MNPs as nucleic acid carriers.

**Table 2:** Advantages and limitations of MNPs for nucleic acid delivery

Types	Advantages	Limitations	References
Gold	▪ Ability to regulate their size ▪ Complementary biocompatibility ▪ Shape and surface functionalization on the nano and molecular level ▪ Safety	▪ Difficulty of control the size and morphology	[117, 128]
Silver	▪ Antibacterial	▪ Genotoxicity ▪ Non-specific biological toxicity	[66, 128–130]
Iron oxide	▪ Biodegradability	▪ Nonreproducibility of the synthesis ▪ Agglomeration of the colloidal suspension	[131, 135, 144]
Magnetic	▪ Possibility for selective target site treatment ▪ Simple monitoring ▪ Assistant drug or gene release	▪ Toxicity ▪ Agglomeration (pH 7) ▪ Difficulty of control the size and morphology	[136–139]
Silica	▪ Capable of conjugating with almost ▪ all types of functional groups ▪ Biocompatibility	▪ Hemolysis ▪ Toxicity ▪ Difficulty of synthesis	[133–137]
Zinc oxide	▪ Anticancer activity	▪ Cytotoxicity	[155–159]
Copper oxide	▪ Surface and superior quantum size effect ▪ Volume effect and macroscopic quantum tunneling have effects in magnetic and chemical activity ▪ Optical absorption ▪ Thermal resistance ▪ Catalysis and the melting point	▪ Chemical methods synthesize suffer from the adsorption of toxic chemicals	[160–165]
Titanium	▪ Cellular uptake profile and stimuli-responsive	▪ Cytotoxicity	[166, 167]
Selenium	▪ Antioxidant in human health ▪ Superior biocompatibility ▪ Degradability in vivo	▪ Toxicity ▪ Biocompatibility	[168–171]
Palladium	▪ High porosity ▪ Photocatalytic activity ▪ Thermal and chemical stability	▪ Toxicity	[172–175]
Platinum	▪ Anticancer activity	▪ Cytotoxicity ▪ Low biocompatibility	[149, 176–178]

### AuNPs

Among known siRNA delivery systems that researchers studied, AuNPs are alluring due to their extraordinary optical hallmarks compared to other inorganic carriers, such as IONs, silica NPs, and quantum dots. Their relative immobile, complementary biocompatibility is accompanied by the ability to regulate their size, shape, and surface functionalization at the nano and molecular levels [115]. AuNPs have demonstrated their effectiveness for nucleic acid delivery. Several AuNP platforms for nucleic acid delivery, such as amino acid-functionalized AuNPs (AA–AuNPs), mixed-monolayer-protected AuNPs (MM-AuNPs), and layer-by-layer-fabricated AuNPs (LbL-AuNPs) have shown stability and safety [116]. Klibanov and Thomas reported that Polyethyleneimine (PEI)–AuNP, as a delivery vector of pDNA, showed higher efficiency than PEI counterparts in monkey kidney (COS-7) cells [117]. First-generation lysine dendron (G1-Lys)–coated AuNPs showed effective gene delivery of plasmid double-stranded DNA in COS-1 cells [118]. AuNPs stabilized with mercaptoundecanoic acid and coated with layers of PEI and siRNAs provided stable nucleic acid delivery and controlled nucleic acid release in CHO-K1 cells [119]. The anticancer effect was observed on PEI-Au/human telomerase reverse transcriptase (hTERT) siRNA and PEI-Au/hTERTsiRNA@ZGOC samples in B16F10 cells (murine melanoma cell line) [120]. It is possible to control the structural property of AuNPs by experimental methods, however it is still potentially expensive due to the requirement of distinguishing structural control of the wide range of size, shape, and surface chemistry [121].

Shayu et al. designed multifunctional Aptamer-tethered DNA-Au particle (Apt-DNA-Au) nanomachine to serve as a therapeutic as well as diagnostic nanoplatform for targeted therapy. It acts as a fluorescence imaging-guided chemogenic, photodynamic, and photothermal synergistic system in breast cancer. This nanomachine demonstrated great biostability, biocompatibility, and high loading capacity. In vitro and in vivo studies for Thymidine Kinase1 (TK1) mRNA, Apt-DNA-Au nanomachine sensitivity and specificity, have attained real-time monitoring for the dynamic change during the therapy in tumors [122]. Beha et al. introduced multi-layer coated AuNPs (MLGNPs) which target the resistance gene of methicillin-resistant *Staphylococcus aureus* by delivering Antisense Oligonucleotides (ASOs). They prepared MLGNPs with AuNPs, polyethyleneimine, and ASO. Their result showed 74% expression of mecA gene silencing, and, in presence of oxacillin, bacterial growth was also reduced by approximately 71%. They concluded that MLGNPs could be applied to silence a resistance gene for targeting different types of multidrug-resistant bacterial infections [123]. The prostaglandin transporter (PGT) gene overexpression is promoted by hyperglycemia, and it causes poor vascularization and wound healing. In treating diabetic wounds in a type 1 diabetes-induced rat model, the Pluronic F127 (PF127) gels containing DsiRNA-AuNPs appeared to be beneficial. At the end of treatment, gels containing DsiRNA-AuNPs promoted angiogenesis and subsequent wound healing, related to endogenous molecules [Prostaglandin E2 (PGE2) and Vascular Endothelial Growth Factor (VEGF)] levels, verified by Enzyme-Linked Immunosorbent Assay (ELISA). On Day 14 of treatment, new blood vessels and VEGF-A were detected, and the efficacy of DsiRNA in silencing the PGT gene was revealed, resulting in enhanced angiogenesis. Also, PF127 gels containing DsiRNA-AuNPs prevent and inhibit both Gram-positive and Gram-negative bacterial infections. Consequently, the PF127 gel containing DsiRNA-AuNPs-HLRE (F3) presented a greater perspective as a diabetic wound healing promoter in future therapeutic management [124].

Combined with polyethyleneimine Au nanorod that targets cancer-specific cells by arginylglycylaspartic acid (RGD) peptide could condense miRNA to self-assemble supramolecular NPs. These NPs delivered miR-320a specifically and efficiently to lung cancer cells. In vitro and in vivo, combined with laser irradiation, Au-RGD-miR-320a NPs noticeably suppressed proliferation and metastasis, and promoted apoptosis of lung cancer. By directly binding to the 3sed prolife (3’ untranslated region of stimulating protein 1), miR-320a suppresses Sp1 expression, and ultimately the expression of phosphatase and tensin homolog (PTEN) was promoted, inhibiting the expression of Matrix Metallopeptidase 9 (MMP9) [125].

From a recent perspective, MNPs can be considered promising components that can overcome the hurdles of conventional therapy and imaging system. Likewise, AuNPs (smaller size gold) can usually be better to cross the BBB through the spaces among astrocyte capillary endothelium and end-feet, pass to the brain tumor tissue, and reach a more homogeneous intra-tumoral distribution [126, 127]. One study by Huang et al. described that AuNPs smaller than 10 nm could distribute throughout the cellular cytoplasm and nucleus of cancerous cells, while larger size NPs could be located only in the cytoplasm, where they have formed aggregates [127]. The clinical trials also showed that spherical AuNPs (NU-0129) act as gene carriers, having a crucial role in treating late-stage cancers by regulating Bcl2L12 gene expression levels (Clinical Trials: NCT03020017) [128].

### AgNPs

AgNPs are broadly studied for their potential antibacterial properties. Nano-sized AgNPs affect both Gram-positive and Gram-negative microbes [130]. Either chemically or biologically synthesized, AgNPs have even shown anticancer properties [131]. AgNPs for a delivery system can be synthesized by several methods, such as chemical reduction, evaporation–condensation, and green synthesis [65]. Synthesized Arg-Gly-Asp-Ser motif (RGDS) decorated with Chitosan-graft-Poly(acrylamide) (PEG/CTS-g-PAAm)@AgNPs can be used as an efficient carrier of the gene due to a higher ability to complex with DNA [80]. AgNPs with a carbosilane-dendrons-modified surface form stable complexes with siRNA, ensuring efficient cellular uptake by protecting it from enzymatic degradation [117]. However, the biomedical applications of AgNPs are limited by their genotoxicity toward mammalian cell and their non-specific biological toxicity [130].

### IONs/magnetic NPs

Nanotechnology provides a flexible platform for advancing efficient therapeutic nanomaterials in a biological system that can behave specifically with a target, and arouse an expected biological response. IONs have become, among the top candidates, one of the most well-studied nanomaterials for cancer therapy. Due to its inherent superparamagnetism characteristic enables non-invasive MRI and appropriate biodegradability for in vivo applications [132]. Functionalization of the IONs surface represents one of the key features to develop these materials as drug and gene delivery carriers [133]. Superparamagnetic IONs (SPIONs) have been used due to the magnetic hyperthermia mechanism for cancer therapy, and can be targeted directly to tumor cells [134, 135]. Due to their remarkably low toxicity, ability to immobilize biological materials on their surfaces, low cost of production, and potential for direct targeting using external magnets, SPIONs have attracted outstanding attention for gene delivery applications [136]. On the contrary, the non-reproducibility of the synthesis and clusterization of the colloidal suspension has restrained SPIONs. To overwhelm these challenges, the surface of the SPION should be sheltered with natural or synthetic polymers [134].

Magnetic NPs have been extensively used in bio applications. For example, cell separation, hyperthermia, drug or gene delivery, ultrasensitive detection, simultaneous imaging, targeted therapy of tumors, and real-time monitoring of drug distribution in vivo [137]. They offer several advantages: (a) high-cell transfection with the probability for selective target area treatment via high-field/high-gradient magnets (magnetofection); (b) superparamagnetic properties of MNPs provide simple monitoring by using MRI; (c) can enhance restorative power through boosting drug or gene release, or ablation of tumors in tissues by hyperthermia under a magnetic field; (d) multi-functionality including biological labeling/imaging, magnetic separation, and delivery for drug or gene therapeutics [138]. A substantial improvement in the kinetics of the delivery process, the dose–response relationship in nucleic acid delivery, and the nucleic acid delivery localization possibility to an area under magnetic field effect are significant advantages of magnetofection [139]. Magnetic NPs cluster easily in aqueous solutions around pH 7, and it is tough to control the properties and amounts of clustered Magnetic NPs. Their high surface could explain the considerable toxicity of MNPs to volume ratio compared to other microparticles [140].

Moreover, several NP-mediated short nucleotide chain (RNA) delivery systems have been formulated for the glioma gene therapy techniques, directing a range of genes: Bcl2L12, c-Met, epidermal growth factor receptor (EGFR), Polo-like kinase-1 (PLK1), miRNA-21, VEGF, special AT-rich sequence-binding protein-1 (SATB1 protein), and Galectin-1 [141–143]. Researchers also studied different types of NPs (e.g. metal nanostructures, polymeric NPs and LNPs) that are pragmatically used for optimal delivery of siRNAs in pancreatic cancer therapy, both for pre-clinical and clinical applications [144]. Subsequently, studies also established that SPIONs could also act as the radiosensitizer in cancer therapy [145]. Apart from drug and gene delivery, diagnostic function, the MNPs can act as effective molecular imaging agents [146, 147].

### Silica NPs

According to the literature, silica NPs can be classified into three groups, regarding their sizes: microporous (pore size < 2 nm), mesoporous (between 2 and 50 nm), and macroporous (> 50 nm) [148].

Silica-based NPs, especially mesoporous silica NPs (MSNPs), were the first to be introduced by Vallet-Regi et al. in early 2000. They emphasized MSNPs as favorable candidates for tumor-targeted drug and gene delivery through their broad advantages in all types of nanocarriers. The MSNPs surfaces are affluent with reactive silanol groups capable of binding with nearly all types of functional groups, such as polymers, targeting ligands, metal/metal oxide, and fluorescent agents. Designing MSNPs with these functional groups may impart numerous exciting properties such as tumor targeting, bio-imaging, stimuli-responsive release, etc. Additionally, MSNPs have excellent biocompatibility [149]. Indeed, the United States Food and Drug Administration (USFDA) approved silica as an adjuvant (e.g., as an anticaking agent, emulsifier), and silica is “generally regarded as safe” in the food industry [150]. Montalvo-Quiros et al. showed that MSNPs offered a great advantage while carrying the AgNPs by preventing their aggregation and improving the possibility of selective cell targeting and reduced toxicity [151].

Some researchers have examined the new platform for delivery vectors. Fibrous Programmable nucleic acid NPs (NANPs) containing siRNAs for GFP were delivered to human breast cancer cells. In the MDA-MB-231 cell line, expression of GFP was silenced via nucleic acid MSNPs (NA-MSNPs). NANPs were complexed with RNA duplexes against GFP. In their experiments, NA-MSNPs dependent silencing efficacy was related to the shapes of NANPs. Higher knockdown efficiency of 54% and 68% was observed for fibrous (fNANPs) and DS RNAs, while globular (cNANPs) and planar (rNANPs) silencing efficiencies against GFP were 40 and 33% [152].

### Zinc oxide NPs

The potent anticancer action of zinc oxide (ZnO) NPs is considered due to their tumor pH-dependent ion disassociation and the suppression of several kinases. It is also responsible for reactive oxygen species (ROS) generation in nanomedicine which is essential in cancer cell signaling [153]. Zn is a component of nearly 300 enzymes, a vast number of proteins, and a crucial nutrient for many physiological functions such as cell growth, DNA synthesis, bone metabolism, and immune function. Including trigger glucose metabolism and extracellular‐signal‐regulated kinases 1 and 2 (ERK1/2) activation, Zn imitates many activities of insulin [154]. ZnO nanomaterials have been considered prospective photosensitizers for photodynamic therapy due to their unique phototoxic reaction upon ultraviolet (UV) light and demonstration of significant cell-killing results in cancer therapy [155]. ZnO also has a high isoelectric point, nontoxicity, and biocompatibility and is a broadly used material in nanowire biosensor research [156, 157]. Oxidative stress-mediated cell death can be caused by zinc ions generated from ZnO materials, and intense interaction between free Zn ion concentrations. ZnO NPs-caused cytotoxicity also recommends a ZnO dissolution for effective cytotoxicity. The ZnO caspase-dependent apoptotic activity chips might be the consequence of zinc ions’ potential in down-regulating protein levels of anti-apoptotic molecules involving inhibitor of aptotosis proteins (IAP)s, p-p53, claspin, and hypoxia inducible factor 1 subunit alpha (HIF-1α) in Raji cells (human cell line of hematopoietic origin) and also inducing oxidative stress [158].

Yin et al. reported the use of a highly magnetic zinc-doped iron oxide (ZnFe_2_O_4_) core and a biocompatible mesoporous silica (mSi) shell, forming a multifunctional magnetic core–shell NP (MCNP). They used MCNP to overcome chemo-resistance in breast cancer by simultaneously delivering let-7a microRNA and DOX (an anticancer drug). let-7a can suppress DNA repair mechanisms BRCA1, BRCA2, and drug efflux pumps. By delivering let-7a, it can sensitize MDA-MB-231 (chemoresistant breast cancer cell line) to succeed in DOX chemotherapy [159].

In another study, Chen et al. synthesized ZnO microflowers with four distinctive hydrothermal methods at low temperatures in the absence of any surfactant. Scanning electron microscopy was used to characterize the diameters, morphologies, and “petals” of flower-like ZnO. PEI is the most widely used cationic polymer and has low transfection efficiency due to weak DNA binding competence. ZnO microflowers were used to enhance the transfection efficiency of low molecular weight PEI (PEI1.8 k) by forming complexes of PEI1.8 k/pDNA. Experiments analyzing cellular uptake demonstrated that the “petals” of ZnO microflowers penetrated the surface of the cells, assisting gene delivery into the cells. The researchers projected the ZnO microflowers as a favorable adjuvant for effective gene delivery. However, the ZnO microflowers’ cytotoxicity is a little bit disappointing [160].

### Copper oxide (CuO) NPs

For centuries, copper metal complexes have attracted great attention regarding their pharmacological and biological properties, basically due to their many antimicrobial activities [161, 162]. They are also used as an anti‐fungal agent when integrated in plastics, coatings, textiles, etc. [163]. Nano CuO has remarkable physical and chemical attributes like volume effect, surface effect, the advantage of the quantum size effect, macroscopic quantum tunneling potency in a magnetic field, chemical activity, optical absorption, thermal resistance, melting point, and the catalysis contrast compared to typical CuO. Nanometer CuO particles’ sizes are between 1 and 100 nm [164]. To produce CuO NPs, numerous synthetic approaches have been used, such as electrochemical processes, microwave irradiation, and chemical reduction. However, chemical approaches to synthesized CuO NPs deteriorate due to the adsorption of toxic chemicals upon their surface, making them inappropriate for biological applications [165]. Though, several green chemical methods are being explored for their synthesis. Green chemical methods are favored over traditional methods as usually they are non-toxic, cost-effective, and eco-friendly [166].

### Titanium oxide (TiO_2_) NPs

TiO_2_ NPs can be marked as one of the most favorable candidates for biomedical applications due to their outstanding advantages such as low cost, biosafety, nontoxicity and biocompatibility, and their widespread usage in daily life nanocarriers for drug and gene delivery usages. TiO_2_ NPs have drawn much attention in biomedical and cancer applications, which can be mainly attributed to the chemical and physical properties of these nanomaterials [167].

The polymeric LbL miR708/paclitaxel (PTX)/silica-supported mesoporous titaniaNPs (MTNst) efficiently encapsulated miR708 and PTX as combined chemotherapeutic agents. MTNst exhibits greater release at low pH conditions, imitating pH conditions of a tumor. These nano-therapeutic agents showed excellent in vitro cytotoxicity, cellular uptake profiles, and stimuli-responsive delivery against HCT-116 and DLD-1 colorectal carcinoma cells, compared with free PTX or PTX-loaded NPs [168].

### Selenium (Se) NPs

Se is an essential trace element that is able to scavenge free radicals in the body, and to prevent oxidative damage to DNA. From animal health and nutrition to human nutrition, selenium research has advanced from disease prevention and treatment to drug and gene delivery. Some studies have shown that elemental selenium is less toxic compared with its other forms and has outstanding biological activity [169].

SeNPs are considered superior to MNPs like gold, silver, and platinum NPs, due to their unique biocompatibility and degradability in vivo. By virtue of their unique properties, SeNPs have now manifested as a novel form of selenium, finding a place in medicine as delivery vehicles [170]. Positively charged peptide RGDfC-SeNPs (R-SeNPs) were synthesized as tumor-targeted siRNA delivery carriers. Later they were complexed with myocyte enhancer factor 2-D subtypes (MEF2D)-siRNA (R-Se@MEF2D-siRNA). R-Se@MEF2D-siRNA showed low toxic side effects and indicated significant antitumor activity targeting ovarian cancer in mice [171]. The hyaluronic acid-Se-PEI (HA-Se-PEI) NPs was developed as a carrier to deliver HES5-siRNA. Delivered HA-Se-PEI@siRNA showed an anticancer effect due to the silence of the HES5 gene and excellent compatibility in this gene delivery system in vivo [172].

### Palladium (Pd) NPs

The cost of PdNPs is low and has advantages like high porosity, photocatalytic activity, thermal and chemical stability, etc. [173]. It can also be synthesized and modified in a variety of sizes and shapes [174, 175]. Porous PdNPs that are attached with the fluorescein-labeled thiolated DNAzymes (FAM-Dz) for silencing the HCV NS3 gene have been shown to have good gene loading and releasing abilities [176]. However, more research on applications of PdNPs for gene delivery systems is needed.

### Platinum (Pt) NPs

PtNPs are new agents mostly used for drug delivery in cancer diagnosis and therapy. PtNPs have anticancer activity and can improve through functionalization (such as targeting ligand attached-PtNPs) [177]. However, PtNPs can induce cytotoxicity by breaking the DNA strands inside cells [178, 179]. Therefore, there is a lack of research data on PtNPs for nucleic acid delivery. This will continue to limit its use in medicine and healthcare as long as significant issues to improve the safety and biocompatibility of PNPs remain. Thus, robust designing and testing of PtNPs as nucleic acid carriers is required [150], in order to establish them as a good delivery system with high efficiency, safety, and commercial application.

### Metal–organic framework (MOF)

MOF systems can be used for drug delivery in cancer therapy owing to the advantages like adjustable surface and porosity [180]. MOF systems can also be designed for improving the ability of biocompatible, biodegradable, controllable release and cell/tissue targeting [181]. MOF has been widely studied in diverse fields, including delivery systems. Nevertheless, the use of MOF is limited due to certain drawbacks like high costs, difficult regeneration, and low capacity [182].

Cell-penetrating peptides (CPPs)-modified zeolite imidazolyl skeletons (ZIFs) can be used as an effective gene carrier in HeLa cells [183]. ZIF usually demonstrates efficient drug loading and releasing competence. However, the safety and biocompatibility of ZIFs are still unclear and need further studies [184]. Platelet membrane coated MOF/siRNA can specific-bind to the cancer cells, and reduce survivin (overexpressed in most breast carcinomas) expression level in human SK-BR-3 breast cancer cells and in vivo [185]. miR-34a-m@ZIF-8 induces cancer cell apoptosis by suppressing the Bcl-2 expression levels and inhibits the tumor growth in of triple-negative breast cancer mouse model [186]. pH-responsive released Chlorin e6 (Ce6)-DNAzyme@ZIF-8 NPs induced ROS-mediated cell apoptosis with DNAzyme-mediated inhibition of human early growth response-1 in MCF-7 cells (breast cancer cell line) and MCF-7 tumor-bearing BALB/c female nude mice [187]. Biocompatible iron (3) carboxylate MOFs as RNA nanocarriers increased cellular uptake and gene activity in vitro [188]. Bovine Serum Albumin (BSA)-decorated MOF/anti-TNF-α siRNA showed high therapeutic efficacy against rheumatoid arthritis in collagen-induced arthritis (CIA) mice model [189].

### Doped metal materials

He et al. reported that gadolinium (Gd)-doped carbon dots (CDs) indicated blue or green fluorescence in HeLa cells when observed with a confocal laser scanning microscope, suggesting Gd-doped CDs can monitor the gene delivery process and can be used for bioimaging [190]. Lanthanide-doped hollow NPs (LDHNs), as gene delivery vehicles, protected the pDNA (containing enhanced GFP) and increased the transfection efficiency [191]. Fluorescent nitrogen- and zinc-doped CDs (N–Zn-doped CDs) showed excellent transfection efficiency and safe delivery of clustered regularly interspaced short palindromic repeats/Cas9 (CRISPR/Cas9) complexes and mRNA (for degradation of GFP) in HEK 293 T-GFP cells (cells from a kidney human embryo) [192].

### Hybrid nanocariers of MNPs

The inorganic and organic or the bioactive components can be combined into a single material as NPs, creating completely novel and advanced compositions. They have unique properties for drug or gene delivery approaches within the cellular level and are considered hybrid nanocarriers [193]. Specifically, such NPs are developed from the bulk metallic components and show superior chemical functionalization, imparting them with many new properties for therapeutic applications, specifically the delivery of nucleic acids. The multifunctional hybrid nanoplatforms of MNPs have shown desired therapeutic characteristics for targeted agent delivery (e.g., siRNA) [194]. Despite different structural variations, they show an acceptable range of size, site-specificity, and drug and gene-carrying capacity of hybrid nanocarriers. The hybrid nanocarriers reflect novel components with the synergistic effects of diagnostics and therapeutics [195, 196].

Conversely, several challenges still exist for the large-scale development of hybrid nanocarriers precisely for nucleic acids delivery, which might be promising and have enhanced theranostics applications. Anastassacos et al. showed an easy, operative, and scalable chemical cross-linking method to develop stable DNA nanostructures [197]. Furthermore, to ensure the competitiveness of DNA nanostructures over existing nanocarriers and conjugation with the organic and inorganic materials, it can also be planned to expand the stability and specificity to attenuate immune response or allow the distribution via added routes. An exceptional array of hybrid structures can be invented using the modularity of the DNA nanostructures [198, 199]. In addition, the hybrid systems of DNA nanocarriers and proteins can be comprehensive to accumulate multiple proteins, construct enzyme cascades and construct biochips or biosensors [200]. This versatile tool might improve gene delivery, transform the solubility and biological activity, and stabilize therapeutic cargos elements or the nucleic acids-based nanostructure [201, 202]. Ryu et al. lately described a specialized modular design to accumulate functional DNA nanostructures to be used as drug and gene delivery platforms [203]. Zhang et al. also reported developing a DNA nanostructure to deliver a specialized antisense peptide conjugated nucleic acid against methicillin-resistant bacterial (*S. aureus*) infections [204].

## Conclusions and future direction

Nucleic acids as biopharmaceuticals have an immense potential for treating several diseases, including genetic abnormalities, infectious diseases, metabolic, neurological, and musculoskeletal disorders, and even cancers. Nucleic acid drugs are either based on DNA or non-coding RNAs [ASOs, miRNAs, and siRNAs or mRNAs]. Aptamers and CpG oligonucleotides are also considered nucleic acid drugs. Nucleic acid delivery to the targeted cells is necessary for nucleic acids’ efficient and desirable efficacy as drug candidates. However, disadvantages like highly hydrophilic character, polyvalent anionic properties, and large size make cell nucleic acid uptake more undesirable. Few past decades have witnessed substantial growth in nanomedicine, specifically in drug delivery systems. A number of delivery systems have been developed to deliver nucleic acids, which have immensely enhanced the possibilities of their delivery to the targeted cells. Unlike gene therapy, nucleic acid drugs would be required to be administered in a time and dose-dependent manner, largely depending on the patient's clinical manifestation in a particular situation.

With the advent of nanotechnology, innovative approaches are in constant development for the delivery of drug molecules for a variety of purposes. Moreover, novel nanoformulations are being developed even for diagnostics. Gene therapy has the potential to be an attractive therapeutic approach for a number of untreatable diseases, involving both viral or non-viral delivery methods. Non-viral carrier systems are favored over viral vectors due to their efficient biological barriers crossing ability. Nanocarriers promise to achieve the potential of gene therapy by enabling the targeted delivery of nucleic acids to the desired site of action. However, several issues like targeted delivery, biodegradability, cytotoxicity, transfection ability, and size of particles need to be further resolved, to make the nanocarriers more efficient and achieve the desired goals of clinical applications.

**Fig. 4:**
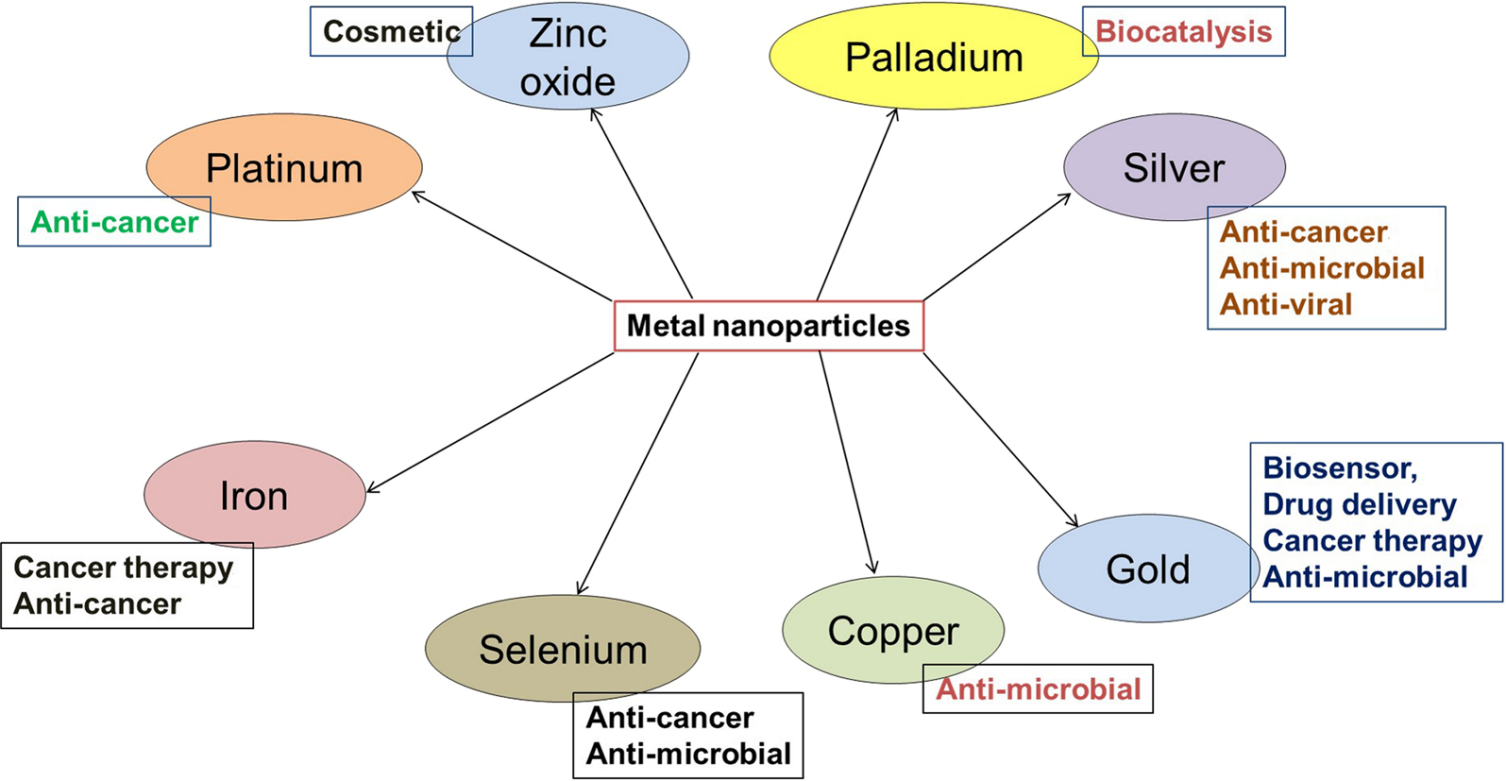
Different types of MNPs and their applications

MNPs are a unique class of nanocarriers for therapeutic delivery of drugs and many other applications (Fig. 4). Properties like well-characterized structure, tunable pore size, porosity, ultrahigh surface area, and chemical functionalization provide them with unique properties for targeted delivery of drugs in clinical settings. However, MNPs are at the early stages of development, and still require refinements to achieve the ability to deliver the therapeutic molecules (including nucleic acids) to the desired site in clinical settings. With the ongoing efforts, it is expected that a few of the discussed applications of MNPs will appear in the near future in clinical trials and then in clinical applications.

## Data Availability

Not applicable.

## Abbreviations

NPs: Nanoparticles
MNPs: Metal-based nanoparticles
AuNPs: Gold nanoparticles
AgNPs: Silver nanoparticles
IONs: Iron oxide NPs
BBB: Blood–brain barrier
PEG: Polyethylene glycol
DOX: Doxorubicin
CB-CNP: Cathepsin B-sensitive nanoprobes
MRI: Magnetic resonance imaging
DNA: Deoxyribonucleic acids
RNA: Ribonucleic acid
PLGA/FPL NPs: Poly(lactide-co-glycolide)/folate coated PEGylated polymeric liposome core–shell nanoparticles
LNPs: Lipid-based nanoparticles
CAR: Chimeric antigen receptor
pDNA: Plasmid DNA
RPE: Retinal pigment epithelia
CHO: Chinese hamster ovarian
LMWP: Low molecular weight protamine
PD: Peptide-pDNA
LPD: Liposome-peptide-pDNA
GFP: Green fluorescent protein
PMOXA: Polymer poly (2-methyl-2-oxazoline)
PASP: Peptide block poly (aspartic acid)
DET: Diethylene-triamine
PPH: Polymer–peptide hybrid
PAMAM: Poly(amidoamine)
Cur: Curcumin
ABP: Arginine grafted bio-reducible polymer
hMSC: Human mesenchymal stem cell
CMC: Critical micelle concentration
PM: Polyplex micelles
CA-CSO-SA: Cis-aconitate-modified chitosan-g-stearic acid
AA–AuNPs: Amino acid-functionalized gold nanoparticles
MM–AuNPs: Mixed-monolayer-protected gold nanoparticles
PEI: Polyethyleneimine
G1-Lys: First-generation lysine dendron
hTERT: Human telomerase reverse transcriptase
Apt-DNA-Au: Aptamer-tethered DNA-Gold particle
TK1: Thymidine kinase1
MLGNPs: Multi-layer coated gold nanoparticles
ASOs: Antisense oligonucleotides
PGT: Prostaglandin transporter
PF127: Pluronic F127
PGE2: Prostaglandin E2
VEGF: Vascular Endothelial Growth Factor
RGD: Arginylglycylaspartic acid
PTEN: Phosphatase and tensin homolog
MMP9: Matrix metallopeptidase 9
RGDS: Arg-Gly-Asp-Ser motif
PEG/CTS-g-PAAm: Polyethylene glycol/chitosan-graft-poly(acrylamide)
SPIONs: Superparamagnetic iron oxide nanoparticles
EGFR: Epidermal growth factor receptor
PLK1: Polo-like kinase-1
SATB1 protein: Special AT-rich sequence-binding protein-1
MSNPs: Mesoporous silica nanoparticles
FDA: Food and drug administration
NANPs: Nucleic acid nanoparticles
NA-MS-NPs: Nucleic acid mesoporous silica nanoparticles
fNANPs: Fibrous nucleic acid nanoparticles
cNANPs: Globular nucleic acid nanoparticles
rNANPs: Planar nucleic acid nanoparticles
ZnO: Zinc oxide
ROS: Reactive oxygen species
ERK1/2: Extracellular‐signal‐regulated kinases 1 and 2
ZnFe_2_O_4_: Zinc-doped iron oxide
UV: Ultraviolet
IAPs: Inhibitor of aptotosis proteins
HIF-1α: Hypoxia inducible factor 1 aubunit alpha
mSi: Mesoporous silica
MCNP: Magnetic core–shell nanoparticle
CuO: Copper oxide
TiO_2_: Titanium oxide
PTX: Paclitaxel
MTNst: Silica-supported mesoporous titania nanoparticles
Se: Selenium
MEF2D: Myocyte enhancer factor 2-D subtypes
HA-Se-PEI: Hyaluronic acid-selenium-polyethyleneimine
PdNPs: Palladium nanoparticles
FAM-Dz: Fluorescein-labeled thiolated DNAzymes
PtNPs: Platinum nanoparticles
MOF: Metal–organic framework
ZIF-8: Zeolite imidazolyl skeleton-8
BSA: Bovine serum albumin
CIA: Collagen-induced arthritis
CPPs: Cell-penetrating peptides
Gd: Gadolinium
CDs: Carbon dots
LDHNs: Lanthanide-doped hollow namoparticles
N–Zn-doped CDs: Nitrogen- and zinc-doped carbon dots
CRISPR/Cas9: Clustered regularly interspaced short palindromic repeats/Cas9
